# Increasing Ciliary ARL13B Expression Drives Active and Inhibitor-Resistant Smoothened and GLI into Glioma Primary Cilia

**DOI:** 10.3390/cells12192354

**Published:** 2023-09-26

**Authors:** Ping Shi, Jia Tian, Julianne C. Mallinger, Dahao Ling, Loic P. Deleyrolle, Jeremy C. McIntyre, Tamara Caspary, Joshua J. Breunig, Matthew R. Sarkisian

**Affiliations:** 1 Department of Neuroscience, University of Florida College of Medicine, Gainesville, FL 32610, USA; ivy.c.shi@gmail.com (P.S.); tian.jia@ufl.edu (J.T.); jmallinger1118@gmail.com (J.C.M.); dling1@ufl.edu (D.L.); jmcin@ufl.edu (J.C.M.); 2 Preston A. Wells Jr. Center for Brain Tumor Therapy, University of Florida College of Medicine, Gainesville, FL 32610, USA; loic.deleyrolle@neurosurgery.ufl.edu; 3 Department of Neurosurgery, University of Florida College of Medicine, Gainesville, FL 32610, USA; 4 Department of Human Genetics, Emory School of Medicine, Atlanta, GA 30322, USA; tcaspar@emory.edu; 5 Board of Governors Regenerative Medicine Institute, Cedars-Sinai Medical Center, Los Angeles, CA 90048, USA; joshua.breunig@cshs.org; 6 Department of Biomedical Sciences, Cedars-Sinai Medical Center, Los Angeles, CA 90048, USA; 7 Samuel Oschin Comprehensive Cancer Institute, Cedars-Sinai Medical Center, Los Angeles, CA 90048, USA

**Keywords:** primary cilia, hedgehog, glioblastoma, GLI2, smoothened, vismodegib, cyclopamine, INPP5e, IFT88

## Abstract

ADP-ribosylation factor-like protein 13B (ARL13B), a regulatory GTPase and guanine exchange factor (GEF), enriches in primary cilia and promotes tumorigenesis in part by regulating Smoothened (SMO), GLI, and Sonic Hedgehog (SHH) signaling. Gliomas with increased *ARL13B*, *SMO*, and *GLI2* expression are more aggressive, but the relationship to cilia is unclear. Previous studies have showed that increasing ARL13B in glioblastoma cells promoted ciliary SMO accumulation, independent of exogenous SHH addition. Here, we show that SMO accumulation is due to increased ciliary, but not extraciliary, ARL13B. Increasing ARL13B expression promotes the accumulation of both activated SMO and GLI2 in glioma cilia. ARL13B-driven increases in ciliary SMO and GLI2 are resistant to SMO inhibitors, GDC-0449, and cyclopamine. Surprisingly, ARL13B-induced changes in ciliary SMO/GLI2 did not correlate with canonical changes in downstream SHH pathway genes. However, glioma cell lines whose cilia overexpress WT but not guanine exchange factor-deficient ARL13B, display reduced INPP5e, a ciliary membrane component whose depletion may favor SMO/GLI2 enrichment. Glioma cells overexpressing ARL13B also display reduced ciliary intraflagellar transport 88 (IFT88), suggesting that altered retrograde transport could further promote SMO/GLI accumulation. Collectively, our data suggest that factors increasing ARL13B expression in glioma cells may promote both changes in ciliary membrane characteristics and IFT proteins, leading to the accumulation of drug-resistant SMO and GLI. The downstream targets and consequences of these ciliary changes require further investigation.

## 1. Introduction

Glioblastoma (GBM) is the most lethal form of primary brain tumors in adults, presumably originating from a transformed glial cell lineage [1]. GBMs also evolve from lower grade gliomas [2,3,4]. GBMs inevitably recur and the average patient survival from diagnosis averages 15–18 months despite standard-of-care therapies (surgery, temozolomide (TMZ) chemotherapy, and irradiation) [5,6]. Tumors have complex modes of signaling that allow them to escape therapy and promote malignancy, including changes in gene expression patterns and signaling pathway alterations. How cellular structures contribute to these signaling pathways remains unclear. The primary cilium is a relatively understudied organelle in gliomas, and a major signaling structure of many cell types, both normal and malignant [7]. Cilia concentrate several signaling pathways, the most well-characterized being sonic hedgehog (SHH) [8]. SHH is one of three types of vertebrate hedgehog (Hh) ligands that stimulate cell proliferation, survival, and migration of normal and cancer cells [9,10]. While the SHH pathway appears overactive in a significant fraction of GBMs [11,12,13,14,15], little research has been done to understand the relationship of SHH and cilia in low- or high-grade glioma.

GBM tumors and derived cell lines from patients can display up to ~30% ciliated cells at any given time [16,17,18,19,20,21]. Like other cell types, glioma cilia enrich ARL13B [18,20], a regulatory GTPase and GEF localized to the ciliary membrane and required for ciliary structural maintenance [22]. Recent studies suggest ARL13B is critical for tumor growth in vitro and in vivo, as well as for angiogenesis within GBM [18,19,23,24,25]. For example, mice carrying intracranial GBM tumors expressing dominant-negative KIF3A lacked ARL13B^+^ cilia and displayed prolonged survival [17]. Knockdown of ARL13B within all GBM subtypes slowed intracranial tumor growth in vivo [25]. More recently, signaling through or at ARL13B^+^ cilia appears critical for cells to maintain a proliferative or stem-like state through engagement of the SHH pathway. For example, histone deacetylase 6 (HDAC6) inhibition can trigger glioma stem cell differentiation by suppressing SHH signaling [26]. However, this phenomenon is not observed in glioma cells depleted of ARL13B [23]. Similarly, KLHDC8a stimulates glioma stem cell growth by promoting ciliogenesis and SHH pathway activation. Inhibition of ARL13B with shRNA or ciliogenesis using ciliobrevin slowed tumor growth and reduced stem cell markers and SHH pathway activation [19]. Thus, ARL13B appears to play a key role in regulating GBM growth and maintaining glioma stem cells.

ARL13B is a mediator of SHH signaling in the cilium, as loss or mutation of ARL13B alters the enrichment of SHH signaling components in cilia along with downstream transcriptional signaling [27,28]. Canonically, SHH binds its receptor, Patched (i.e., *PTCH1* and *PTCH2* genes), on the ciliary membrane and the complex is trafficked to the cell. The G protein-coupled receptor smoothened (SMO) is then visibly enriched in cilia, where it is activated presumably by lipid components in the ciliary plasma membrane such as cholesterol, oxysterols, phosphoinositides, endocannabinoids, and arachidonic acid derivatives [29,30,31]. SMO activation drives the activation of GLI transcription factors, notably GLI1 and GLI2 [32,33], resulting in transcriptional changes in the nucleus [34,35]. This pathway is inhibited by blocking processes leading to the activation of SMO, including mutations altering cilia function [36]. However, knockout of *Arl13b* in a mouse model results in constitutive SMO ciliary enrichment and SHH ligand-independent pathway activation [22,27,37]. Further, a single point mutation, V358A, prevents ARL13B from enriching in cilia [28,38], but preserves its GTPase and GEF activity [38]. *Arl13b^V358A/V358A^* mice are viable, and surprisingly display normal SHH signaling and ciliary SMO enrichment [38]. Thus, how these findings relate to glioma cells and whether the relationship between ARL13B and SMO is SHH-dependent remains unclear.

Hyperactive forms of SMO signaling and ciliary localization are oncogenic [9], and thus, SMO signaling must be tightly controlled. The Cancer Genome Atlas (TCGA) data sets indicate that gliomas with high *ARL13B* and *SMO* mRNA expression correlate with shorter overall patient survival [18,25]. Whether high *ARL13B* and *SMO* expression are coincidental or related is not clear. Our previous findings implicated a relationship between ARL13B and SMO in glioma cells that may, in part, be independent of SHH. In one of our GBM-patient-derived cell lines (L0) expressing *Arl13b:GFP* transgenes [18], we observed that glioma cells displayed elongated cilia, accumulation of SMO at their ciliary tips, and excision of their distal tips. This SMO accumulation in *Arl13b:GFP* transgene-expressing cells occurred in the absence of SHH stimulation and was as robust as SHH-stimulated parental cells [18]. However, we do not know whether the ARL13B-mediated changes in the glioma cilia length or the SMO distribution are attributable to ARL13B’s ciliary or cellular role. Here, we used additional cell lines and known mutations that affect ARL13B subcellular localization and GEF activity. Our goal was to try and distinguish whether ARL13B inside or outside of glioma cilia drives the ciliary changes in SMO, and whether ARL13B drives increases in activated ciliary SMO.

## 2. Materials and Methods

### 2.1. Cell Culture

Human U138 MG (Cat #HTB-16), U87 MG (Cat #HTB-14), and A172 (Cat #CRL-1620) and rat F98 (Cat #CRL-2397) cells were all obtained from ATCC (Gaithersburg, MD, USA) and maintained in recommended media conditions. Mouse KR158 glioma cells were derived from a murine grade III anaplastic astrocytoma [39] and maintained in DMEM ([-] sodium pyruvate; Corning; Cat #10017CV), 10% heat-inactivated fetal bovine serum (FBS) (Atlanta Biologicals, Flowery Branch, GA USA; Cat #SH30070.03), and 1% penicillin–streptomycin. L0 (grade IV glioblastoma from a 43-year-old male) and S7 (grade II glioma from a 54-year-old female with EGFR amplification) cell lines were isolated and maintained as previously described [20,40,41,42]. R24-03 and R24-06 cell lines were expanded from a 91-year-old male and 63-year-old male GBM, respectively. L0 and S7 cells were grown as floating spheres and maintained in NeuroCult NS-A Proliferation medium and 10% proliferation supplement (STEMCELL Technologies, Vancouver, BC, Canada; Cat #05750 and #05753), 1% penicillin–streptomycin (Thermofisher, Waltham, MA, USA; Cat #15140122), 20 ng/mL human epidermal growth factor (hEGF) (Cat #78006), and 10 ng/mL basic fibroblast growth factor (bFGF) (STEMCELL Technologies; Cat #78003). For S7 cells, the media was supplemented with 2 μg/mL heparin (STEMCELL Technologies; Cat #07980). When cells reached confluency, or spheres reached approximately 150 μm in diameter, they were enzymatically dissociated by digestion with Accumax (Innovative Cell Technologies, San Diego, CA, USA; Cat #AM-105) for 10 min at 37 °C. For human cells grown on glass coverslips, NeuroCult NS-A Proliferation medium was supplemented with 10% heat-inactivated fetal bovine serum (FBS) (Cytiva, Marlborough, MA, USA; Cat #SH30070.03HI). NIH3T3 cells (ATCC, Gaithersburg, MD, USA; Cat #CRL-1658) were maintained in DMEM (ATCC; Cat #30-2002), 10% FBS, and 1% penicillin–streptomycin. All cells were grown in a humidified incubator at 37 °C with 5% CO_2_. Where indicated, indicated cell lines were treated with various drugs including cyclopamine (Calbiochem, San Diego, CA, USA; Cat #239803) (diluted in dimethyl sulfoxide (DMSO)), temozolomide (TMZ) (Sigma, St. Louis, MO, USA; Cat #T2577) (diluted in DMSO), GDC-0449 (Cayman Chemical, Ann Arbor, MI, USA; Cat #13613), SAG (Calbiochem; Cat #566661), and recombinant human SHH (high-activity) (R&D Systems, Minneapolis, MN, USA; Cat #8908-SH-005/CF), and fixed after indicated treatment durations and processed as described below. Where indicated, cells were also transiently transfected with pFlag-Smo (PD22) (gift from G. Pazour), pCAG-Ar13b^WT^:GFPpb, and pDest-Arl13b^T35N^:GFP cDNA using Lipofectamine 3000 (Invitrogen, Carlsbad, CA USA; Cat #L3000-008), and fixed at the indicated timepoints as described below.

S7 clones stably expressing mouse *Arl13B^WT^:GFP* or *Arl13B^V358A^:GFP* were generated using piggybac transposase similarly to previously described methods [18]. Briefly, S7 cells were grown as adherent in 6-well plates and transfected at 60-70% confluence with a total of 500 ng per well of pCAG-pBase and pCAG-Arl13b^WT^:GFPpb or pCAG-ARL13B^V358A^:GFP vectors using 10 µL of Lipofectamine 3000. To generate the pCAG-Ar13b^WT^:GFPpb or pCAG-Ar13b^V358A^:GFPpb vector, we subcloned the C-terminal GFP-tagged Arl13b sequence from pDest-Arl13b WT:GFP or pDest-Arl13b^V358A^:GFP into the pCAG-pb vector. Approximately 1 week after transfection, individual GFP^+^ clones were sorted and expanded in 96-well plates each containing 250 μL of supplemented S7 medium without FBS using a BD FACS Aria II Cell Sorter (BD Biosciences, San Jose, CA, USA). Cell debris were excluded from the analysis by forward- and side-scatter gating. Subsequently, expanded GFP^+^ clones were propagated.

### 2.2. Western Blot

Western blot was performed as recently described [23]. Briefly, cells were harvested at indicated time points and lysed in 1× cell lysis buffer (Cell Signaling, Danvers, MA, USA; Cat #9803) or 1× radioimmunoprecipitation assay (RIPA) buffer (Cell Signaling, Danvers, MA, USA; Cat #501015489) containing 1× protease inhibitor cocktail (Sigma, St. Louis, MO, USA; Cat #P2850), phosphatase inhibitor cocktails 1 (Sigma, St. Louis, MO, USA; Cat #P5726) and 2 (Sigma; Cat #P0044), and 1× phenylmethanesulfonyl fluoride (Sigma, St. Louis, MO, USA; Cat #93482). Prior to loading, samples were heated to 70 °C for 10 min. A total of 25–30 μg of total protein lysate per lane was separated on 4–12% Bis-Tris gels (Thermofisher; Cat #NP0050). Proteins were blotted onto PVDF membranes using iBlot (program 3 for 8 min; Invitrogen, Carlsbad, CA, USA). Blots were blocked in 5% non-fat dry milk (NFDM) or bovine serum albumin (BSA, Jackson Immuno Research, West Grove, PA, USA; Cat #NC9871802) in 1× tris-buffered saline (TBS) with 0.1% Tween (TBST) for 20 min and then incubated in primary antibodies in 2.5% NFDM or BSA in 1× TBST for 24 h at 4 °C. Blots were rinsed and probed in the appropriate horseradish peroxidase (HRP)-conjugated secondary antibody (1:10,000; BioRad, Hercules, CA, USA) for 30 min at RT in 2.5% NFDM or BSA in 1× TBST. Finally, blots were rinsed in 1× TBS and developed using an Amersham ECL chemiluminescence kit (Global Life Sciences Solutions USA, Marlborough, MA, USA), and images were captured using an AlphaInnotech Fluorchem Q Imaging System (Protein Simple, San Jose, CA, USA). Selected areas surrounding the predicted molecular weight of the protein of interest were extracted from whole blot images.

### 2.3. Bulk RNA Sequencing

S7 cells were transfected with pCAG-pBase and either pCAG-Ar13b^WT^:GFPpb or pCAG-Ar13b^V358A^:GFPpb vectors. Control cells were mock transfected (no cDNA). Flow cytometry was used to isolate single control or GFP^+^ cells in 96-well plates which were then expanded to generate nine S7 cell lines (three per group: control, WT, or V358A). Offline data were analyzed on the University of Florida High-Performance Cluster (HiPerGator). Short reads were trimmed using trimmomatic (v0.36) [43] and QC on the original, and trimmed reads were performed using FastQC (v0.11.4) [https://www.bioinformatics.babraham.ac.uk/projects/fastqc/ (accessed on 6 April 2023] and MultiQC [44]. The reads were aligned to the transcriptome using STAR (v2.7.9a) [45]. Transcript abundance was quantified using RSEM (v1.3.1) [46]. Differential expression analysis was performed using DESeq2 [47], with an FDR-corrected *p*-value threshold of 0.05. The output files were further filtered to extract transcripts showing a 1.5-fold change in either direction. Results were reported for protein-coding genes only, and for all transcript types.

### 2.4. Immunocytochemistry

For immunocytochemical analyses, samples were fixed at indicated timepoints with 4% paraformaldehyde in 0.1 M phosphate buffer (4% PFA) for 15 min and washed with 1× PBS. Cells were immunolabeled for the indicated primary antibodies (Table 1). Samples were incubated in blocking solution containing 5% normal donkey serum (NDS) (Jackson Immunoresearch, West Grove, PA, USA; Cat #NC9624464) and 0.2% Triton-X 100 in 1× PBS for 1 h and then incubated in primary antibodies with 2.5% NDS and 0.1% Triton-X 100 in 1× PBS either for 2 h at room temperature (RT) or overnight at 4 °C. Appropriate FITC-, Cy3- or Cy5-conjugated secondary antibodies (1:1000; Jackson ImmunoResearch) in 2.5% NDS with 1× PBS were applied for 1–2 h at RT, and coverslips were mounted onto Superfrost^TM^ Plus coated glass slides (Fisher Scientific, Cat #12-550-15) in Prolong Gold antifade media containing DAPI (Thermofisher; Cat #P36935). Stained coverslips were examined under epifluorescence using an inverted Zeiss AxioObserver D1 microscope using a Zeiss 40×/0.95 plan Apochromat air objective or a Zeiss 63×/1.4 plan Apochromat oil objective. Images were captured and analyzed using Zeiss ZEN software (ZEN 2012 (Blue edition) v1.1.2.0).

For analyses of cilia length, ARL13B^+^ cilia were traced using a line measurement tool in Zeiss ZEN software. For analyses of immunofluorescence staining intensity, areas were traced around the cell cilium and the mean fluorescence intensity (MFI) was background corrected. The background MFI, measured from an area between the cells, was subtracted from the cilia MFI. To determine percent IFT88^+^ cilia, for each image we divided the number of cilia with clear IFT88 puncta at the tip by the total number of either ARL13B^+^ (parental) or ARL13B^WT^:GFP^+^ (transgenic) cilia. Overall, we analyzed cilia from at least 2–4 coverslips per group.

### 2.5. Data Analysis

Statistical analyses were performed using GraphPad Prizm 9.0 (GraphPad Software, La Jolla, CA, USA). In all analyses, *p* values less than 0.05 were considered significant. Comparisons between groups were performed using either a Student’s *t* test or one-way analysis of variance (ANOVA) followed by Tukey’s post hoc analysis, as indicated. To compare the distribution of Smo-Flag localization, we used a Fisher’s Exact test.

## 3. Results

### 3.1. ARL13B^+^ Cilia Are Present in GBM Biopsies and Recently Derived Patient Cell Lines

Previous studies of widely used GBM cell lines (e.g., U-lines), found that primary cilia are typically absent, structurally impaired, or rarely elongated [48]. We immunostained these cells to examine the presence of ARL13B^+^ cilia. Our experiments support previous findings and extend them to other widely used glioma lines (A172, F98) (Supplementary Figure S1A–D). However, using biopsy material, recently derived low-grade (S7) and high-grade (L0, R24-03, and R24-06) human glioma cell lines, and high-grade mouse glioma cells (KR158) [39], we, and others, find that ARL13B^+^ cilia are readily identifiable [18,20,25,49] (Supplementary Figure S1E–I). Thus, while previous results may represent some types of gliomas, there may be an association of ARL13B and the cilium with other aggressive gliomas.

### 3.2. Increasing ARL13B Expression Correlates with an Increase in SMO and GLI2 and More Aggressive Glioma 

**Figure 1:**
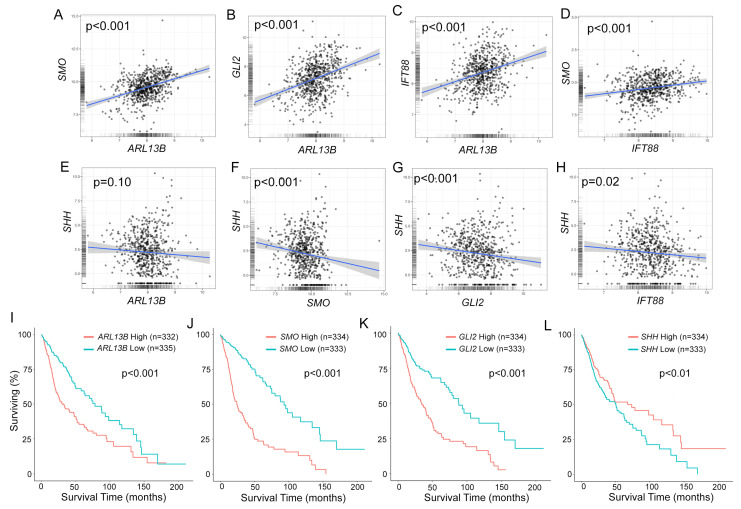
Increasing *ARL13B* expression correlates with *SMO* and *GLI2* and more aggressive glioma. (**A**–**H**) Correlation of gene expressions for indicated gene. *p* values from 2-sided Pearson’s product moment correlation test are indicated. (**I**–**L**) Overall survival curve for the indicated gene when highly (red line) or lowly (blue line) expressed (cutoff = median). *p* values represent log-rank test. All data were extracted from Gliovis database using TCGA for combined low-grade glioma (LGG) and glioblastoma (GBM).

We first examined the relationship of *ARL13B* expression and another key ciliogenesis gene, *IFT88*, with the expression of other SHH pathway genes (SMO, GLI2, and SHH) in low-grade glioma (LGG) and high-grade glioblastoma (GBM) using TCGA datasets. Strikingly, we find a significant positive correlation between *ARL13B* and *SMO*, as well as between *ARL13B* and *GLI2* (Figure 1A,B). Not surprisingly, we observe a positive correlation between *ARL13B* and *IFT88* (Figure 1C). *SMO* also positively correlated with *IFT88* expression (Figure 1D). However, *SHH* expression did not correlate with *ARL13B* (Figure 1E) and was significantly negatively correlated with *SMO*, *GLI2,* and *IFT88* expression (Figure 1F–H).

These correlations are also reflected in overall survival curves of glioma patients. A higher expression of *ARL13B* (Figure 1I), *SMO* (Figure 1J), and *GLI2* (Figure 1K) correlates with significantly shorter survival outcomes. In contrast, higher *SHH* expression correlates with significantly longer survival (Figure 1L). These data suggest that increased *ARL13B* is accompanied by *SMO* and *GLI2*, but not *SHH*, which is surprising as it may be predicted to accompany changes associated with the *SHH* pathway.

### 3.3. Increasing a GFP-Tagged, Functional ARL13B in Glioma Cilia Elongates Them and Promotes SMO Accumulation 

**Figure 2:**
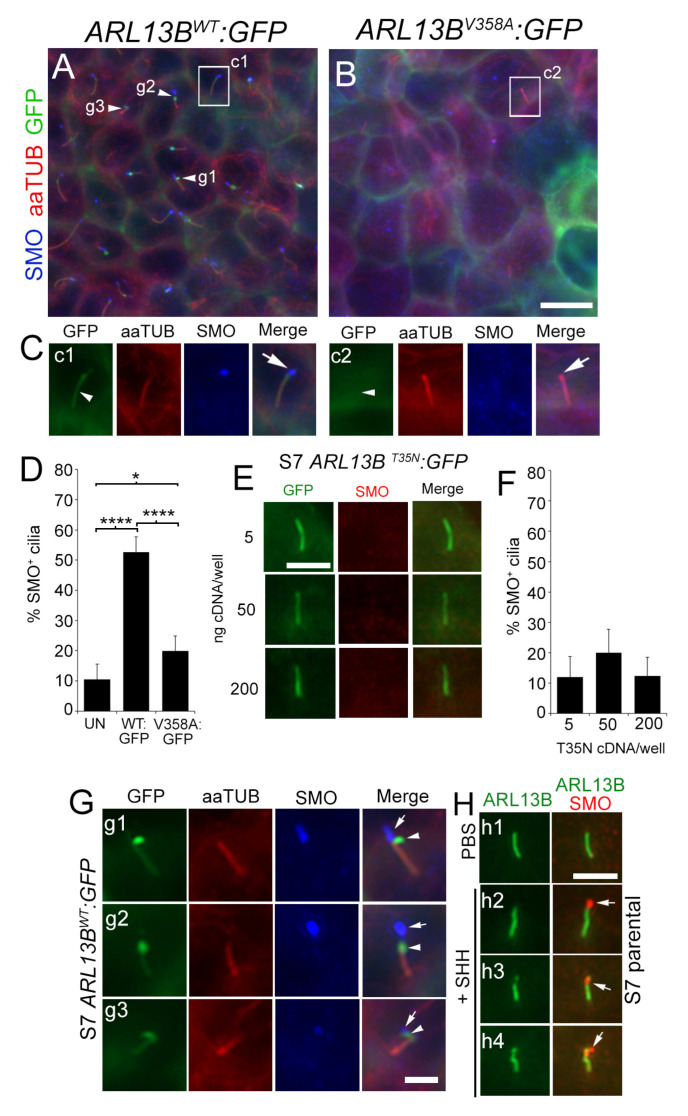
Increasing ciliary ARL13B promotes ciliary SMO enrichment in glioma cilia. (**A**,**B**) S7 transgenic cell lines expressing ARL13B^WT^:GFP (**A**) or ARL13B^V358A^:GFP (**B**). Cells were immunostained for acetylated alpha-tubulin (aaTUB, red) and smoothened (SMO; blue). (**C**) Zoom of boxed areas in (**A**,**B**). ARL13B^WT^:GFP^+^ cilium (arrowhead, **c1**) colocalizes with aaTUB and SMO puncta (arrow). The ARL13B^V358A^:GFP^+^ cilium is positive for aaTUB but lacks GFP (arrowhead, **c2**) and SMO puncta (arrow). (**D**) Bar graph shows % of aaTUB^+^ cilia with SMO^+^ cilia in untransfected (UN) (n = 182 cilia), ARL13B^WT^:GFP^+^ (n = 297 cilia), and ARL13B^V358A^:GFP^+^ (n = 128 cilia) S7 cells. (**E**) S7 parental cells transfected with indicated concentration of *ARL13B^T35N^:GFP* cDNA. Cells were fixed after 48 h and immunostained for SMO (red). Scale bar = 5 µm. (**F**) Quantification of results in E. Bar graph shows % of GFP^+^ cilia with SMO^+^ cilia after 5 (n = 26 cilia), 50 (n = 35 cilia), or 200 (n = 37 cilia) ng of *ARL13B^T35N^:GFP* cDNA/well. (**G**) Three zoomed examples from indicated cilia in (**A**) showing ARL13B^WT^:GFP^+^ cilia in which the GFP (arrowhead) and SMO (arrow) signals appear to dissociate at the distal cilia tip. (**H**) Cilia of parental S7 cells treated with vehicle (PBS) (**h1**) or recombinant human SHH [0.1 ug/µL] (**h2**–**h4**) and fixed after 24 h. SHH-treated cilia display SMO puncta (arrow) that lack or weakly express ARL13B. Scale bars in (**B**) = 10 µm, (**E**,**G**,**H**) = 5 µm. * *p* < 0.05, **** *p* < 0.0001 (ANOVA).

We next examined the effects of increasing ARL13B on glioma cilia morphology and SMO localization. To test whether ARL13B stimulation of ciliary lengthening and SMO accumulation depends on ARL13B levels and function within the cilium, we took advantage of known mutations in ARL13B. The T35N mutation ablates the GEF function and alters the GxP binding of ARL13B but localizes to cilia [37,50], whereas the V358A mutation has normal GEF activity, but is undetectable in cilia [28,38]. Previous studies showed that increasing transfected amounts of ARL13b:GFP cDNA progressively increases protein and cilia length in zebrafish embryos [37], which we confirmed in our cultures (Supplementary Figure S2A,B). Thus, we transfected parental S7 cells with increasing concentrations of GFP-tagged WT, T35N, and V358A plasmid cDNA and immunostained cells for GFP, acetylated alpha tubulin (aaTUB), or ARL13B. On GFP^+^ transfected cells, we measured the length of GFP^+^ cilia (for WT and T35N) or endogenous ARL13B^+^ cilia (for V358A). We found that WT ARL13B:GFP, but not T35N or V358A mutants, significantly elongated cilia as cDNA concentrations increased (Supplementary Figure S2C). We also confirmed that after immunostaining for ARL13B, cells transfected with WT and T35N displayed significantly increased total ciliary ARL13B compared with untransfected cells/cilia (Supplementary Figure S2D). We then generated an S7 cell line stably expressing ARL13B^WT^:GFP and ARL13B^V358A^:GFP using piggybac transgenesis. As expected, GFP expression in ARL13B ^WT^:GFP cells was localized to plasma membranes and cilia (Figure 2A, (Cc1), Supplementary Figure S3A). However, in ARL13B^V358A^:GFP cells, GFP was largely undetectable in cilia but strongly expressed throughout the plasma membranes (Figure 2B, (Cc2), Supplementary Figure S3A). Strikingly, immunostaining revealed significant accumulation of SMO in ARL13B^WT^:GFP cilia tips (Figure 2A, (Cc1), Supplementary Figure S3B), consistent with previous studies in L0 cells [18], that did not occur to the same extent in ARL13B^V358A^:GFP cilia tips (Figure 2B, (Cc2)). Quantification of the percentage of SMO^+^ cilia indicates that ARL13B^WT^:GFP cells display significantly more SMO^+^ cilia than untransfected or ARL13B^V358A^:GFP cells (Figure 2D). We did find that V358A:GFP cells displayed significantly more SMO^+^ cilia than untransfected (Figure 2D), which may be attributable to a weak GFP^+^ signal sometimes observed in the V358A overexpressing cilia [28,38]. Nevertheless, the ARL13B^V358A^:GFP mutant suggests ARL13B inside cilia is stimulating SMO accumulation. Thus, we examined SMO expression while increasing expression of the ARL13B^T35N^:GFP mutant, however, SMO did not significantly accumulate in these mutant cilia (Figure 2E,F).

Closer examination of ARL13B^WT^:GFP-expressing cell cilia suggests that SMO dissociates from ARL13B at the distal cilia tips, giving the appearance that it may be budding off of the cilium (Figure 2G). This phenotype is unlikely to be an overexpression artifact because similar patterns of distal tip puncta that are strongly SMO^+^, but weakly labeled for ARL13B, also occur in parental S7 cells stimulated with exogenous SHH (Figure 2H). Collectively, these data suggest that increasing functional ARL13B inside the cilium leads to the lengthening of glioma cilia and a distal tip accumulation of SMO that is independent of exogenous SHH exposure.

### 3.4. Increasing ARL13B Expression Promotes Abnormal Ciliary Tip Accumulation of Activated SMO

**Figure 3:**
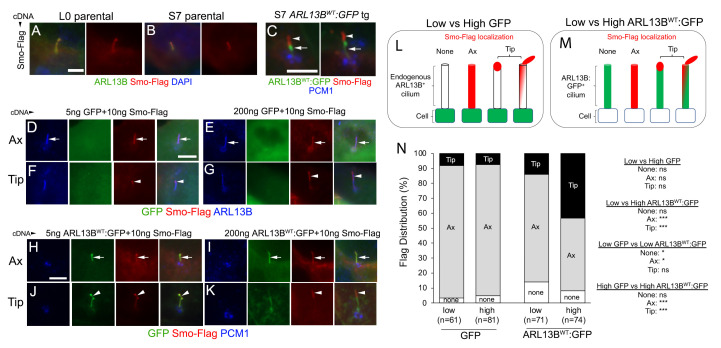
Increasing ARL13B expression promotes ciliary tip accumulation of flag-tagged Smo. (**A**,**B**) L0 and S7 parental cilium expressing wildtype flag-tagged Smo (red) which co-localize along the length of endogenous ARL13B^+^ cilia (arrows). (**C**) S7 ARL13b^WT^:GFP^+^ cilia transfected with flag-Smo shows abnormal Smo accumulation (arrowheads) at the tips of cilia. Immunolabeling for PCM1 (blue) reveals the pericentriolar material that usually concentrates around the ciliary base. (**D**–**G**) Increasing *GFP* cDNA while holding the amount of *Smo-Flag* cDNA constant. cDNA amounts are ng/well. Examples show Smo-Flag localization along the axoneme (Ax) (arrows in (**D**,**E**)) or more asymmetrical accumulations toward the ciliary tip (Tip) (arrowheads in (**F**,**G**)). (**H**–**K**) Increasing *Arl13B^WT^:GFP* cDNA while holding the amount of Smo-Flag cDNA constant. Examples of Smo-Flag localization along the axoneme (Ax) (arrows in (**H**,**I**)) and asymmetric accumulation toward the ciliary tip (Tip) (arrows (**J**,**K**)). (**L**,**M**) Classification of Smo-Flag localization along ARL13B^+^ cilia of GFP^+^ cells (**L**) or ARL13B^WT^:GFP^+^ cilia (**M**). Smo-Flag was either not found in the cilium (None), along the axoneme (Ax), or accumulated/shifted toward or branching off the ciliary tip (Tip). (**N**) Stacked bar graphs show distribution (percent) of Smo-Flag localization in ARL13B^+^ cilia in low vs. high GFP cDNA compared with cilia in low vs. high *Arl13b^WT^:GFP* cDNA. n = number of cilia analyzed. Data were statistically analyzed using a Fisher’s Exact test (* *p* < 0.05, *** *p* < 0.001). Scale bars in (**A**,**C**,**D**,**H**) = 5 µm.

We next asked if increasing ARL13B promotes and accelerates a flag-tagged form of SMO to glioma ciliary distal tips. First, we transfected two different parental glioma cell lines (L0 and S7) with Flag-tagged SMO, which revealed that SMO localized along the axonemal length of endogenous ARL13B (Figure 3A,B). However, in the context of ARL13B^WT^:GFP overexpression, SMO-Flag accumulated at the distal tips (Figure 3C), similar to the endogenous SMO staining in transgenic lines (Figure 2B,C,G). To determine if increasing ARL13B enhances a distal tip accumulation of SMO, we transfected low (5 ng/well) or high (200 ng/well) concentrations of ARL13b^WT^:GFP plasmid while maintaining a low concentration of FLAG-Smo plasmid (10 ng/well) and stained cells for GFP, FLAG, and PCM1 to label the pericentriolar material around the ciliary basal body. As a control, we transfected low (5 ng/well) or high (200 ng/well) concentrations of GFP plasmid while maintaining a low concentration of FLAG-Smo plasmid (10 ng/well) and stained cells for GFP, FLAG, and ARL13B to label endogenous cilia. In all groups, we observed some transfected cells that lacked Smo-Flag in the cilia (data not shown), or displayed SMO-Flag signal along the ciliary axoneme (Figure 3D,E,H,I) or at the tip (Figure 3F,G,J,K). We then compared the patterns of Smo-Flag localization between low vs. high GFP (Figure 3L) or low vs. high ARL13B^WT^:GFP (Figure 3M). While no significant changes in the distribution pattern of Smo-Flag localization were observed in low vs. high GFP (Figure 3N), higher concentrations of Arl13b^WT^:GFP cDNA promoted a significant increase in the percentage of Smo-Flag tip localization (Figure 3N). Similar shifts in Smo-Flag localization toward the ciliary tip were observed in L0 and R24-3 cell lines when concentrations of Arl13b^WT^:GFP cDNA were increased (Supplementary Figure S4). These results suggest that increasing functional ARL13B in cilia drives SMO toward the ciliary tip.

**Figure 4:**
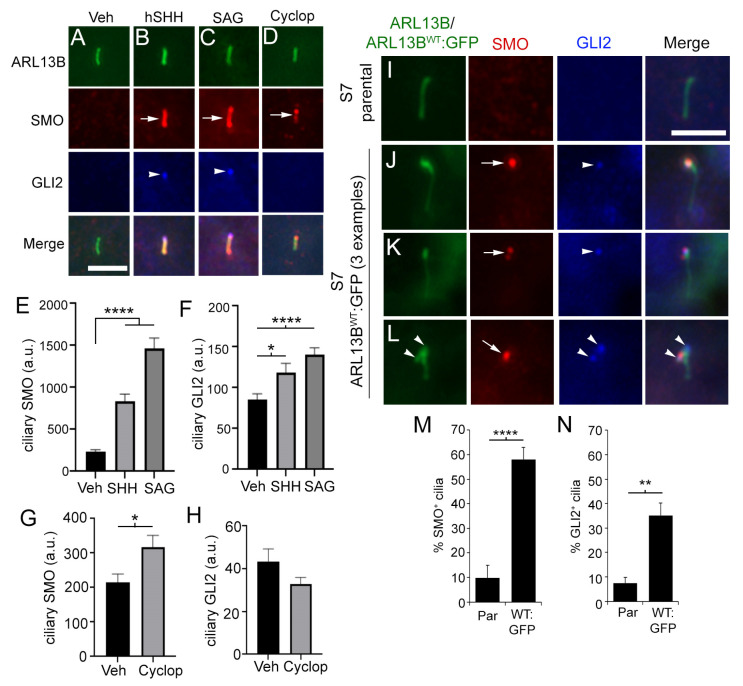
ARL13B overexpression drives active SMO and GLI2 localization in glioma. (**A**–**D**) NIH3T3 cells were treated with vehicle (Veh), 0.1 µg/mL recombinant human SHH, 200 nM SAG, or 5 µM cyclopamine (Cyclop). After 24 h, cells were fixed and triple immunolabeled for ARL13B (green), SMO (red), and GLI2 (blue). (**E**) Quantification of the SMO fluorescence intensity within cilia of untransfected (veh) (n = 39 cilia), SHH (n = 32 cilia), and SAG (n = 44 cilia). (**F**) Quantification of the GLI2 fluorescence intensity within cilia of untransfected (veh) (n = 39 cilia), SHH (n = 32 cilia), and SAG (n = 44 cilia). (**G**) Quantification of the SMO fluorescence intensity within cilia of Veh (n = 37 cilia) or cyclop (n = 42 cilia). (**H**) Quantification of the GLI2 fluorescence intensity within cilia of Veh (n = 37 cilia) or cyclop (n = 42 cilia). (**I**) S7 parental cells immunolabeled for ARL13B (green), SMO (red), and GLI2 (blue). (**J**–**L**) Three examples of ARL13B^WT^:GFP^+^ cilia immunolabeled for SMO and GLI2. Puncta of SMO (arrows) and GLI2 (arrowhead) are localized at the ciliary tips. (**M,N**) Bar graphs show % of endogenous ARL13B^+^ (parental) or GFP^+^ cilia that were SMO^+^ (**M**) or GLI2^+^ (**N**). S7 parental (par) (n = 270) and ARL13B^WT^:GFP^+^ (WT:GFP) (n = 70) cilia. Scale bars in (**A**,**I**) = 5 µm. * *p* < 0.05, ** *p* < 0.01, **** *p* < 0.0001 (ANOVA).

It is unclear if the accumulated SMO in glioma cilia in ARL13B-overexpressing cells is in an active or inactive state. To address the role of ARL13B in promoting active SMO, we used Hh-responsive mouse NIH3T3 cells [34]. Previous studies showed that GLI2 is a key mediator of activated SMO in the cilium leading to transcriptional changes in the nucleus [34,35]. We treated NIH3T3 cells with recombinant human SHH or the SMO agonist, SAG, and found SMO and GLI2 enriched in cilia (Figure 4A–C,E,F). Cyclopamine inhibits SMO activation, yet promotes SMO ciliary enrichment, providing a way to uncouple SMO localization and activation [34,51]. Indeed, we confirmed that cyclopamine stimulated SMO into cilia (Figure 4D,G), but we did not detect GLI2 (Figure 4D,H). Taken together, these data indicate that the coordination of SMO and GLI2 ciliary enrichment is due to activated SMO in cilia recruiting GLI2. Considering GLI2 localization correlated with active ciliary SMO in NIH3T3 cells, we next immunolabeled S7 parental and S7 ARL13B^WT^:GFP cells for SMO and GLI2. Compared with S7 parental cilia that express a low frequency of SMO and GLI2 (Figure 4I,M,N), we observed a significantly higher frequency of ARL13B^WT^:GFP^+^ cilia whose tips were positive for both (Figure 4J–L,M,N), further implicating an accumulation of activated SMO. Altogether, these data support the likelihood that increased ARL13B promotes ciliary enrichment of activated SMO and GLI2.

### 3.5. ARL13B-Induced Accumulation of Ciliary SMO and GLI2 Is Resistant to Potent SMO Inhibitors 

**Figure 5:**
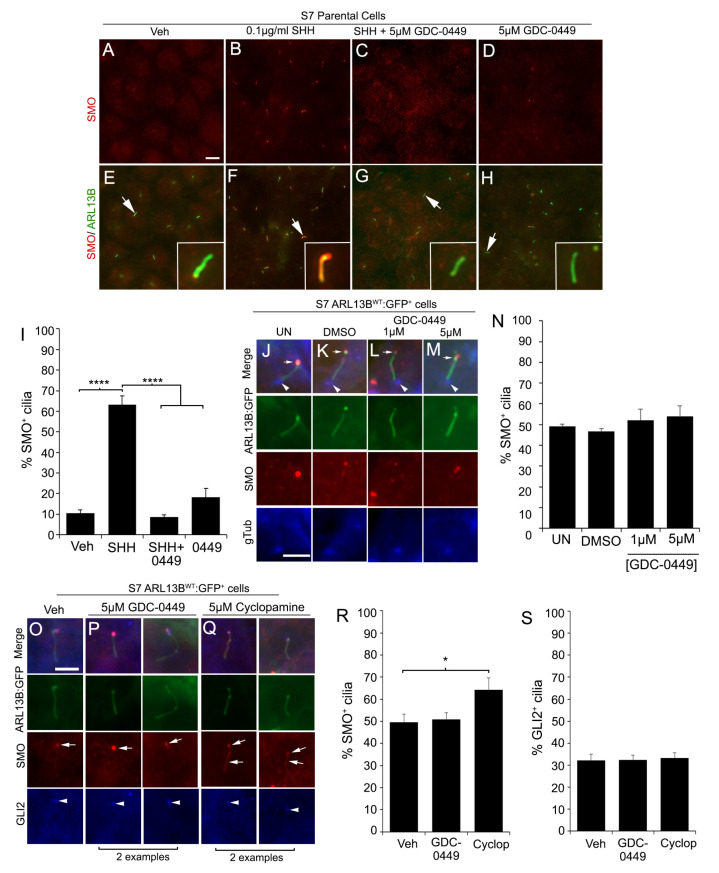
GDC-0449 (Vismodegib) and cyclopamine do not block the ARL13B^WT^:GFP-induced increase in ciliary SMO and GLI2. (**A**–**H**) S7 parental cells treated with PBS (**A**,**E**), 0.1 ug/mL rhSHH (**B**,**F**), rhSHH^+^ 5µM GDC-0449 (**C**,**G**), or 5 µM GDC-0449 (**D**,**H**). After 24 h, cells were fixed and immunolabeled for SMO (red) and ARL13B (green). SHH treatment induces a robust increase in ciliary SMO (arrows in **B**,**F**) in ARL13B^+^ cilia but not in GDC-0449-treated cells. (**I**) Quantification of (**A**–**H**) shows percentage of SMO^+^ cilia in S7 parental cells treated with Veh (n = 100), SHH (n = 249), SHH^+^ 0449 (n = 155), and 0449 (n = 215). (**J**–**M**) S7 ARL13B^WT^:GFP^+^ cells treated with 1 or 5 µM GDC-0449 display SMO (red) at their cilia tips (arrows). The cilia base is immunolabeled with gamma-tubulin (gTub, blue, arrowheads). (**N**) Quantification of (**J**–**M**) shows percentage of SMO^+^ cilia in untreated (UN) (n = 168), DMSO (n = 145), 1 µM (n = 122), or 5 µM (n = 150) cilia. (**O**–**Q**) S7 ARL13B^WT^:GFP^+^ cells treated with veh (**O**), 5 µM GDC-0449 (**P**, 2 examples), or 5 µM cyclopamine (**Q**, 2 examples), fixed after 48 h and immunolabeled for SMO (red) and GLI2 (blue). The cilia/ciliary tips were SMO^+^ (arrow) and GLI2^+^ (arrowheads) in all groups. (**R**,**S**) Quantification of (**O**–**Q**) shows percentage of SMO^+^(**R**) and GLI2^+^ (**S**) cilia in cells treated with veh (n = 534), GDC-0449 (n = 479), or cyclop (n = 461). Scale bars (in µm) in (**A**) = 10, (**J**) = 5, (**O**) = 5. * *p* < 0.05, **** *p* < 0.0001 (ANOVA).

If ARL13B can drive SMO/GLI into cilia, an important question is if this localization can be disrupted by current SMO inhibitors. As we show above and as has been reported by others, cyclopamine promotes ciliary localization of SMO, potentially because the drug alters the conformation of SMO so that it cannot be ubiquitinated and removed from cilia [34,51,52]. To determine whether increased ciliary SMO is due to ARL13B overexpression, we tested other SMO inhibitors that disrupt SMO ciliary localization. In NIH3T3 cells, GDC-0449 (Vismodegib) has been shown to block the SHH-induced increase in ciliary SMO [53]. We confirmed that SHH-stimulated ciliary SMO in S7 parental glioma cells was blocked by pretreatment with GDC-0449 (Figure 5A–H,I). Despite exposure to different concentrations of GDC-0449, we found that ARL13B^WT^:GFP^+^ cilia remain able to accumulate ciliary SMO at their distal tip in the absence of exogenous SHH (Figure 5J–M,N). Treatment with 5 µM GDC-0449 also does not lower accumulation of GLI2 with SMO at the distal tips of ARL13B^WT^:GFP^+^ cilia (Figure 5O,P,R,S). Similarly, SMO and GLI2 accumulation was observed and not reduced after 24 h treatment with 5 µM cyclopamine (Figure 5Q,R,S). These results suggest ARL13B-driven changes in ciliary SMO are resistant to potent pharmacological inhibitors of SMO in glioma cells.

### 3.6. ARL13B:GFP-Induced Changes in Cilia Are Not Associated with Canonical Downstream SHH Pathway Activation 

Considering that ARL13B:GFP induced an accumulation of SMO and GLI2, we asked whether this was associated with downstream changes in the SHH pathway. We first sorted and expanded several additional S7 control and ARL13B^WT^:GFP- and ARL13B^V358A^:GFP-expressing clones and verified the GFP signal (Supplementary Figure S5A–D). We then submitted harvested spheres for bulk RNAseq analysis. However, we were unable to detect any significant changes in the SHH pathway (e.g., SHH, PTCH1, PTCH2, GLI1, GLI2, GLI3, SMO, or SUFU) compared with other genes with high fold changes in the samples (Supplementary Figure S5E,F). Further, the addition of recombinant human SHH to parental S7 cells did not lead to any changes in the SHH pathway (data not shown). Together, these data suggest that increasing ARL13B at/in the cilium alters the SHH transduction pathway components inside glioma cilia, but that downstream pathways either fail to engage in our cells, or that there are non-canonical targets of these transduction events associated with an active SMO/GLI accumulation.

### 3.7. Glioma Cilia Overexpressing WT, but Not T35N ARL13B, Display Reduced Ciliary INPP5e

Since the increase in ciliary SMO/GLI appears to be independent of transcriptional changes in the SHH pathway, we explored whether increases in ciliary ARL13B alter the ciliary membrane properties in a way that could favor SMO/GLI accumulation. Despite being contiguous, the cilia plasma membrane is uniquely composed of phosphatidylinositol 4-phosphate (PI(4)P) compared with the surrounding PI(4,5)P_2_-enriched plasma membrane, a strict compartmentalization which is established by inositol polyphosphate-5-phosphatase E (INPP5e) [54,55]. Recent studies on hTERT-RPE1 cells suggest that INPP5e ciliary membrane retention is dependent on ARL13B [56] and when *ARL13b* is knocked down by siRNA, INPP5e is reduced in cilia [57]. Knockout of *INPP5e* in mouse embryonic fibroblasts dramatically reduces SMO localization [58]. However, mutations of INPP5e (D477N) in neural progenitors of human cerebral organoids were recently shown to reduce INPP5e in ARL13B^+^ primary cilia and result in increased SMO^+^ and GLI2^+^ primary cilia [59]. Thus, we compared INPP5e expression in parental and ARL13B-overexpressing glioma cells.

**Figure 6:**
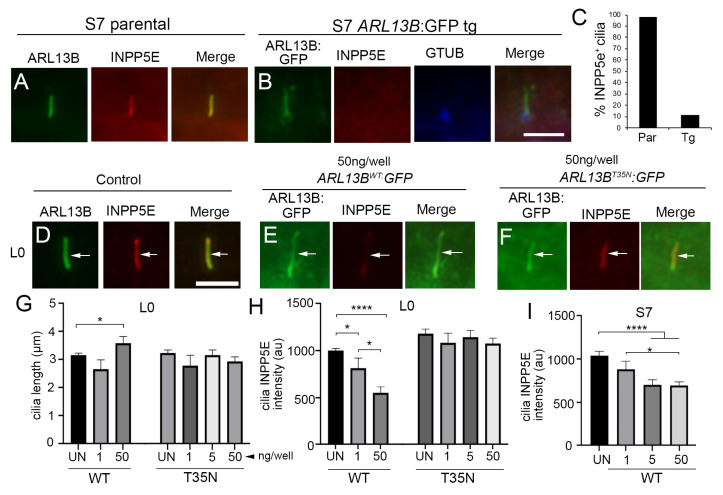
Increasing ARL13B^WT^:GFP decreases ciliary INPP5E. (**A**) S7 parental cell cilium expressing endogenous ARL13B (green) and INPP5E (red). (**B**) Immunostaining of S7 ARL13B^WT^:GFP^+^ cells for INPP5e (red) and gamma tubulin. The GFP^+^ cilium lacks INPP5E. (**C**) Percentage of parental cell cilia (n = 46) with ARL13B^+^/INPP5E^+^ cilia compared with *ARL13b:GFP* transgenic (tg) cells (n = 51) with GFP^+^/INPP5E^+^ cilia. (**D**–**F**) L0 cells untransfected (**D**) or transfected with 50ng/well *ARL13B^WT^:GFP* (**E**) or *ARL13B^T35N^:GFP* (**F**) immunostained for INPP5E (red). (**G**) L0 cilia lengths for indicated group and plasmid concentration per well. (**H**) INPP5E intensity of L0 GFP^+^ cilia in the indicated group. (**I**) INPP5E intensity of S7 GFP^+^ cilia in the indicated group. All intensity analyses were corrected for background. Scale bars in (**B**,**D**) = 5 µm. * *p* < 0.05, **** *p* < 0.0001 (ANOVA).

We first examined INPP5e immunolabeling in the S7 parental and ARL13B^WT^:GFP^+^ transgenic cells and surprisingly found a striking absence of INPP5e^+^ cilia in the transgenic cells (Figure 6A–C). We also examined our L0 cell line, which we previously showed accumulates SMO in ARL13B^WT^:GFP^+^ cilia (18). We varied the amount of transfected ARL13B^WT^:GFP cDNA per well which, as expected, leads to cilia elongation at higher concentrations (Figure 6G). Notably, increased ARL13b^WT^:GFP cDNA corresponded to a reduction in INPP5e in the GFP^+^ cilia (Figure 6D,E,H). We did not observe changes in cilia length or ciliary INPP5e intensity with increasing concentrations of ARL13B^T35N^:GFP cDNA (Figure 6F–H). Increasing ARL13b^WT^:GFP cDNA also correlated with reduced INPP5e in S7 GFP^+^ cilia (Figure 6I). These data suggest that increasing functional ARL13B inside glioma cilia alters ciliary membrane constituents that promote SMO/GLI accumulation.

### 3.8. Glioma Cilia Overexpressing ARL13B^WT^:GFP Display Reduced IFT88

**Figure 7:**
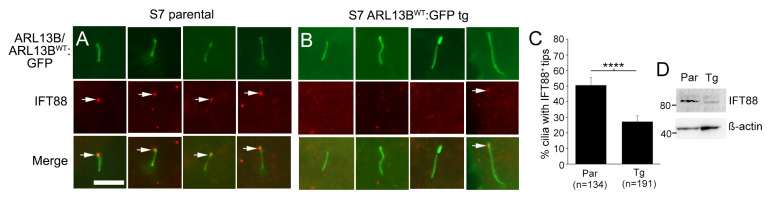
ARL13B^WT^:GFP overexpression reduces ciliary IFT88. (**A**) Four examples of S7 parental cilia displaying endogenous ARL13B (green) and IFT88 (red) at the cilia tip (arrows). Scale bar = 5 µm. (**B**) Immunostaining of S7 ARL13B^WT^:GFP^+^ cells for IFT88. IFT88^+^ puncta at the cilia tip (arrow) were less frequent than control. (**C**) Percentage of S7 parental cell cilia and *ARL13b^WT^:GFP* transgenic (Tg) cells with IFT88^+^ cilia tips. **** *p* < 0.0001 (Student’s *t*-test) (**D**) Western blot of cell lysates from S7 parental or ARL13B^WT^:GFP tg cells for IFT88 and ß-actin.

In addition to altered ciliary membrane dynamics, it is also possible that increasing ARL13B^WT^:GFP alters IFT proteins in a manner that impacts retrograde transport from the ciliary tip. IFT88 is a major component of the IFT retrograde complex B and localizes to cilia and ciliary tips [20,60,61,62]. Thus, we immunostained our S7 parental and ARL13B^WT^:GFP^+^ transgenic cells for IFT88. While we readily observed IFT88 at the ciliary tips of parental S7 cells, IFT88 puncta at ARL13B^WT^:GFP^+^ transgenic cell cilia tips were less clear (Figure 7A,B). Quantification revealed a significant reduction in the percentage of cilia with IFT88 (Figure 7C), and we also observed less IFT88 protein in our S7 transgenic lines through Western blot (Figure 7D). Collectively, these data suggest that increasing functional ARL13B inside glioma cilia may additionally alter the retrograde transport mechanisms, promoting further SMO/GLI accumulation.

## 4. Discussion

We find that increasing ARL13B in glioma cilia stimulates ciliary elongation and an increase in SMO/GLI2 accumulation. This appears to be due to a function of ARL13B within cilia, as we do not see the same changes in cilia or SMO/GLI2 accumulation when we increase cellular ARL13B or target a functionless form of ARL13B to cilia. The ciliary ARL13B-driven increase in SMO is likely to be an active form of SMO that recruits GLI2, resembling cells that have been stimulated exogenously with SHH or SAG. The ARL13B-driven increase in SMO and GLI2 appears resistant to SMO inhibitors. However, in our study, changes in ciliary SMO/GLI2 were not linked to downstream canonical pathway activity. Instead, it appears that increasing ARL13B beyond a certain level inside the cilium may alter membrane and IFT properties of the cilia, such as altered INPP5e and IFT88, which favor SMO/GLI accumulation. Considering our previous studies, which showed that glioma cells excise vesicles from their distal tips [18], it is possible the accumulation of active SMO and GLI that exit from cilia in vesicles may be capable of intercellular signaling to other cancer- or unknown recipient host cells, though this latter phenomenon requires further exploration.

### 4.1. ARL13B-Driven Increases in Ciliary SMO: Failed Retrieval of Activated SMO or a Resistance Mechanism in Glioma?

In cells with increased ARL13B expression, there was significant ciliary distal tip accumulation of SMO (Figure 2 and Figure 3). One possibility is that ARL13B somehow drives activated SMO into cilia faster than it can be brought back out of cilia. In IMCD3 cells, activated GPCRs that are not retrieved by intraflagellar transport and BBSome trains are ectocytosed as ciliary vesicles [63,64]. Thus, ARL13B increases in the cilia may enhance SMO accumulation by disrupting the ability of IFT machinery to retrieve activated SMO. Consistent with this possibility, we observed reduced IFT88 in ARL13B^WT^:GFP^+^ cilia (Figure 7). The initial front loading of SMO into cilia may be related to reports on gastric tumor cells which state that ARL13B directly binds and stabilizes SMO [65]. However, we do not know if this potential complex during ciliary entry exists in glioma. If it does exist, it appears to dissociate at the distal tip, based on our immunolabeling of distal tip buds that appear strongly SMO- but weakly ARL13B-labeled (Figure 2), which would suggest other factors are present in the glioma ciliary tip to promote this dissociation. Alternatively, in glioma, ARL13B does not bind or promote SMO ciliary localization.

The ARL13B-driven increases in ciliary SMO and downstream pathway activation appear resistant to both cyclopamine [65] and GDC-0449 (Figure 5), and increasing ARL13B somehow overrides the efficacy of these inhibitors. It is not clear if ARL13B changes the properties of the ciliary membrane so that SMO inhibitors lose their efficacy and stimulate SMO entry. For example, SMO is activated in cilia by membrane phospholipids (oxysterols) that are uniquely concentrated in ciliary plasma membrane [31]. Increases in ARL13B could alter the organization of these phospholipids, since ARL13B is reported to recruit the lipid phosphatase, INPP5e [57]. Indeed, we observed lower INPP5e expression in ARL13b^WT^:GFP^+^ glioma cilia. These observations appear to support recent observations in human neural progenitor cells in which mutations that lower ciliary INPP5e led to increased ciliary SMO and GLI2 [59]. Alternatively, SMO entry into cilia is also promoted by cAMP-PKA pathway stimulation [66]. However, SMO is believed to be a Gi-coupled receptor that decreases cAMP [67,68,69]. In the context of ARL13B overexpression, it could promote entry of other ciliary receptors that stimulate cAMP-PKA and, indirectly, additional SMO. Whatever the mechanism, ARL13B could change the ciliary membrane, other GPCRs, or IFT proteins in the cilium that favor SMO entry/activation and reduces inhibitor efficacy.

A previous study used CRISPR/Cas9 to generate mice that only expressed ARL13B^V358A^ [38]. These transgenic mice only express ARL13B^V358A^ but still form primary cilia in the neural tube. The mutant cells remain capable of SMO trafficking into cilia, and thus, SMO localization to cilia occurs even in the absence of ciliary ARL13B. There are several differences between our studies that may address this discrepancy. In our study, we examined tumor cells endogenously expressing ARL13B with overexpression of WT or mutant variants. Our findings suggest that extra ARL13B in the cilium changes the way SMO localizes in the cilium. Gigante et al. [38] did not examine if ciliary SMO expression changes or increases upon wildtype ARL13B re-expression in the mutant cells. We observed that V358A:GFP cells displayed more SMO^+^ cilia than control (Figure 2D). It is possible our V358A transgenic cells, which also express endogenous ARL13B, enable more SMO to enter cilia. In addition, Gigante et al. [38] examined normal neural progenitor cells with known downstream SHH/SMO activity, whereas our glioma cells lack pathway induction. Future studies could explore whether glioma cells have or develop a failed positive feedback mechanism leading to ciliary accumulation of SMO.

### 4.2. Are Glioma Cilia Non-Responsive or Responding Differently to SHH/SMO?

Despite the ARL13B-induced accumulation of SMO and GLI in cilia, we failed to observe downstream changes in canonical SHH pathway genes. One possibility is that our cells, while capable of mobilizing transduction signals into the cilia, have either lost their sensitivity or have blocked downstream activation of SHH targets. Similar observations have been made in meningioma cells, which display primary cilia and can enrich activating forms of SMO within them but are unable to transduce downstream SHH pathway activation [70]. Similar results were observed in ciliated SKOV3 ovarian cancer cells that enrich SMO in response to SAG stimulation but fail to activate GLI1 [71]. Like meningiomas or certain ovarian cancer cells, there may be gliomas whose cilia can translocate SMO but do not trigger downstream events. Alternatively, glioma cells may lose the ability to transduce over time, or they may drive non-canonical pathways. It is noteworthy that an increased length of primary cilia on various cancer cell lines has been associated with acquisition of drug resistance [72]. Consistent with these findings, ARL13B increased glioma cilia length and SMO accumulation that was resistant to established SMO inhibitors. Thus, glioma cilia may need to be examined before and after treatment paradigms to determine if length/receptor/membrane characteristics correlate with disease progression.

We also observed that in ARL13B^WT^:GFP^+^ cilia, approximately half of the SMO^+^ cilia are also GLI2^+^ (Figure 4M,N and Figure 5R,S). If all SMO were active, one would have expected nearly all SMO^+^ cilia to be GLI2^+^. Since this is not the case, these observations suggest there could be two different populations of cilia in glioma cells, or alternatively two populations of ciliated glioma cells. A recent study on primary human glioblastoma cells found that the frequencies of cilia differ between glioma stem cells and differentiated glioma cells. Glioma stem cells, defined by SOX2 expression, appear to preferentially display primary cilia with activated hedgehog signaling [19]. Because gliomas are notoriously heterogeneous tumors, future studies should determine if glioma stem cells with ARL13B^+^ cilia display different capacities for enriching or activating SMO and GLI.

### 4.3. What Does High Expression of ARL13B, IFT88, and SMO in TCGA Signify?

While there is a direct correlation between *ARL13b* expression and *IFT88* expression in the TCGA for glioma (Figure 1), the nature of this relationship is unclear. Both proteins play roles in ciliogenesis [22,61], and therefore, the increased expression of both genes could reflect changes in cilia frequency in the tumor. However, ARL13B and IFT88 also have documented roles outside of the cilia. IFT88, for example, is required for orientation of the mitotic spindle during mitosis [73]. Therefore, the relationship between higher expression of ARL13B and IFT88 with respect to their expression and function in cilia is less clear. We found that ARL13B:GFP overexpression correlated with reduced ciliary IFT88 (Figure 7). Interestingly, there is evidence supporting the opposing scenario. Loss of *AHI1* leads to reduced ARL13B expression but increased ciliary IFT88 expression in mouse embryonic fibroblasts [60]. Future analyses of patient gliomas should determine whether IFT88 expression is proportionate to the number of ARL13B^+^ cilia in a tumor or dependent on the quantity of ARL13B within each cilium.

High *ARL13b*, *SMO,* and *GLI2* expression correlate with lower patient survival rates in the TCGA (Figure 1) [18,25]. However, this database does not provide information on the ciliation status within tumors. Our results suggest it may be worth determining if shorter patient survival correlates with higher frequencies of ARL13B^+^ and/or SMO^+^/GLI2^+^ cilia. In addition, high *ARL13b*, *SMO,* and *GLI2* expression does not align with SHH expression (Figure 1). We show that a high, relative to a low, expression of SHH predicts longer patient survival, which is counterintuitive given that ARL13B, SMO, and GLI2 mediate SHH signaling [27,38]. The relationship between ARL13B, SMO, and GLI2 may be independent of SHH expression in glioma. In our previous study [18], and in this study (Figure 2 and Figure 3), we find that ARL13B-mediated enrichment of ciliary SMO resembles the effects of stimulating parental cells with SHH. Alternatively, the correlation between ARL13B, SMO, and GLI2 is related to other factors that possibly control *ARL13b* expression in the tumors. We and others find that TMZ stimulates ARL13B [18,21,25,74]. Given this observation, a significant percentage of tumors may increase ARL13B, SMO, and GLI2 in response to TMZ and therefore promote more aggressive tumors. Considering ARL13B-driven SMO appears to be resistant to SMO inhibitors in culture, it may not be surprising to observe that SMO inhibitors fail to control tumorigenesis. Future studies need to determine whether ARL13B-driven changes in SMO/GLI2 cilia affect tumorigenesis or treatment resistance in an in vivo context.

## Figures and Tables

**Table 1:** Primary antibodies used in this study.

Antigen	Host Species	Dilution	Manufacturer/Catalogue #
ADP ribosylation factor 13B (ARL13B)	Rabbit	1:3000	Proteintech; Cat #17711-1-AP
ADP ribosylation factor 13B (ARL13B)	Mouse	1:3000	Abcam; Cat #AB136648
Acetylated alpha Tubulin (aaTub)	Mouse	1:3000	Sigma; Cat #T6793
Beta-actin	Mouse	1:10,000	Sigma; Cat #A5316
Flag	Mouse	1:5000	Sigma; Cat #F-9291
Gamma-tubulin (gTub)	Mouse	1:3000	Sigma; Cat #T6557
Green fluorescent protein (GFP)	Chicken	1:5000	Abcam; Cat #13970
GLI2	Goat	1:500	R&D Systems; Cat #AF3635
Inositol polyphosphate 5-phosphatase (INPP5E)	Rabbit	1:1000	Proteintech; Cat #17797-1-AP
Intraflagellar transport 88 (IFT88)	Rabbit	1:1000	Proteintech; Cat #13967-1-AP
Pericentriolar material 1 (PCM1)	Rabbit	1:3000	Bethyl; Cat #A301150A
Smoothened (SMO)	Rabbit	1:1000	Abcam; Cat #AB38686

## Data Availability

Data supporting the findings within this study are presented within the article and are available from the corresponding author upon reasonable request.

## Supplementary Materials

The following supporting information can be downloaded at: https://www.mdpi.com/article/10.3390/cells12192354/s1, Figure S1: Widely used glioma cell lines lack ARL13B^+^ cilia; Figure S2: Effects of increasing WT and mutant *ARL13B:GFP* cDNA on ARL13B:GFP protein level, cilia length, and ciliary ARL13B intensity; Figure S3: ARL13B^WT^:GFP and ARL13B^V358A^:GFP expression and SMO localization on ARL13b^WT^:GFP^+^ cilia; Figure S4: Increasing ARL13B expression drives flag-tagged Smo toward the ciliary tip; Figure S5: Lack of SHH pathway activation in S7 *ARL13B^WT^:GFP* and *ARL13B^V358A^:GFP* transgenic cell lines.
