# Supplementary material for: Increasing Ciliary ARL13B Expression Drives Active and Inhibitor-Resistant Smoothened and GLI into Glioma Primary Cilia
Source: Cells. 2023 Sep 26;12(19):2354. doi: 10.3390/cells12192354 (PMC10571910; doi:10.3390/cells12192354)
Supplement: Supplementary file 1 [file cells-12-02354-s001.zip › cells-12-02354-supplementary.pdf]

Supplemental Figures, Legends and Whole Blot Images

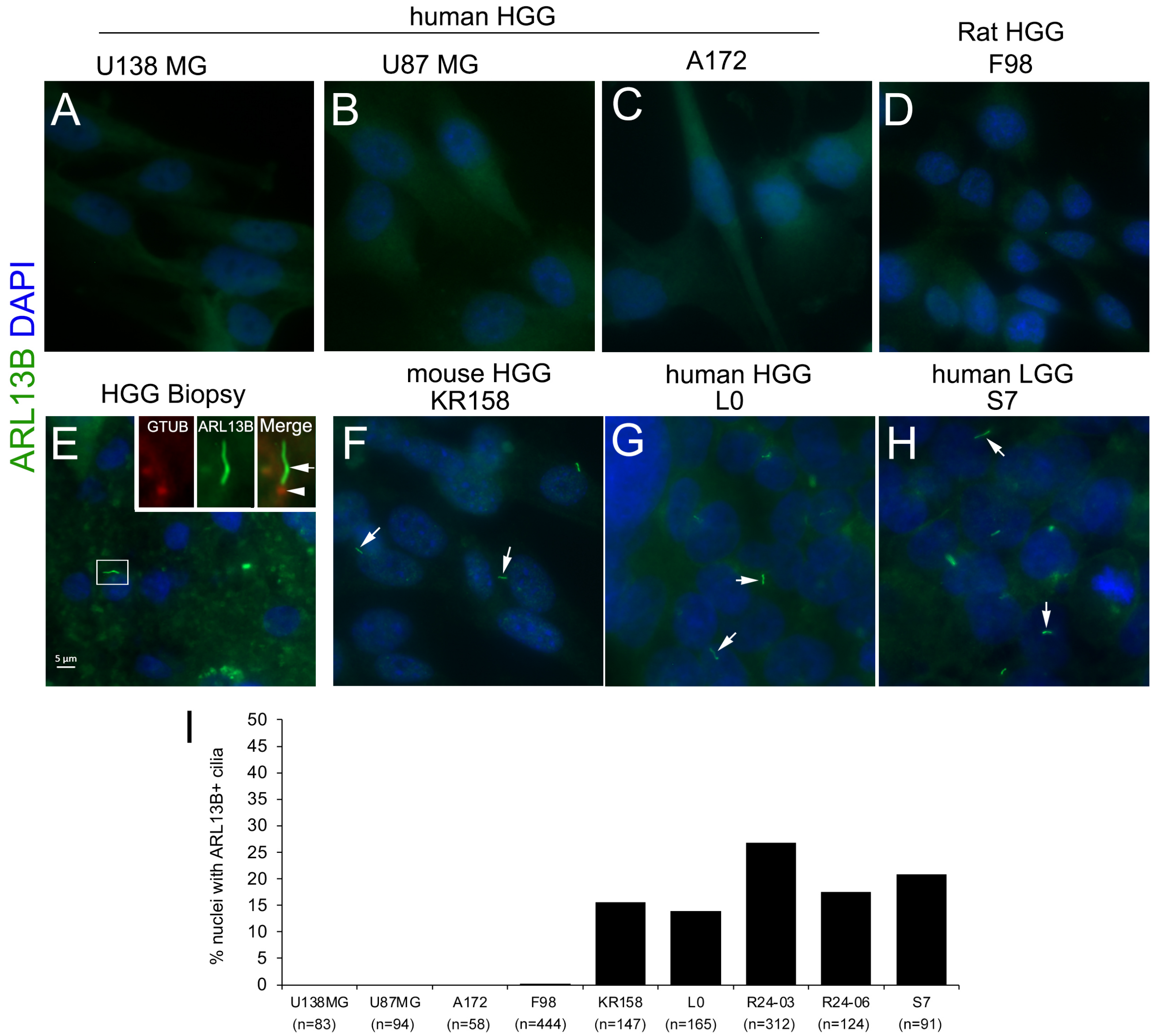

Figure S1. Widely used glioma cell lines lack ARL13B^{+} cilia. **(A-D)** Immunostaining for ARL13B (green) in common human and rat cell lines for GBM/high grade glioma (HGG). Nuclei are blue. **(E)** Example of an ARL13B^{+} cilium (boxed) within a biopsy. Inset shows gamma-tubulin (GTUB, red) staining of the basal body (arrowhead) at the base of an ARL13B^{+} cilium (arrow). (**F-H**) ARL13B^{+} cilia (arrows) observed in mouse HGG **(F)** and human HGG **(G)** and low grade glioma (LGG) **(H).** **(I)** Percent of nuclei in indicated cell line with ARL13B^{+} cilia. n=total # nuclei counted. R24-03 and R24-06 are additional human HGG lines. Scale bar in E = 5µm.

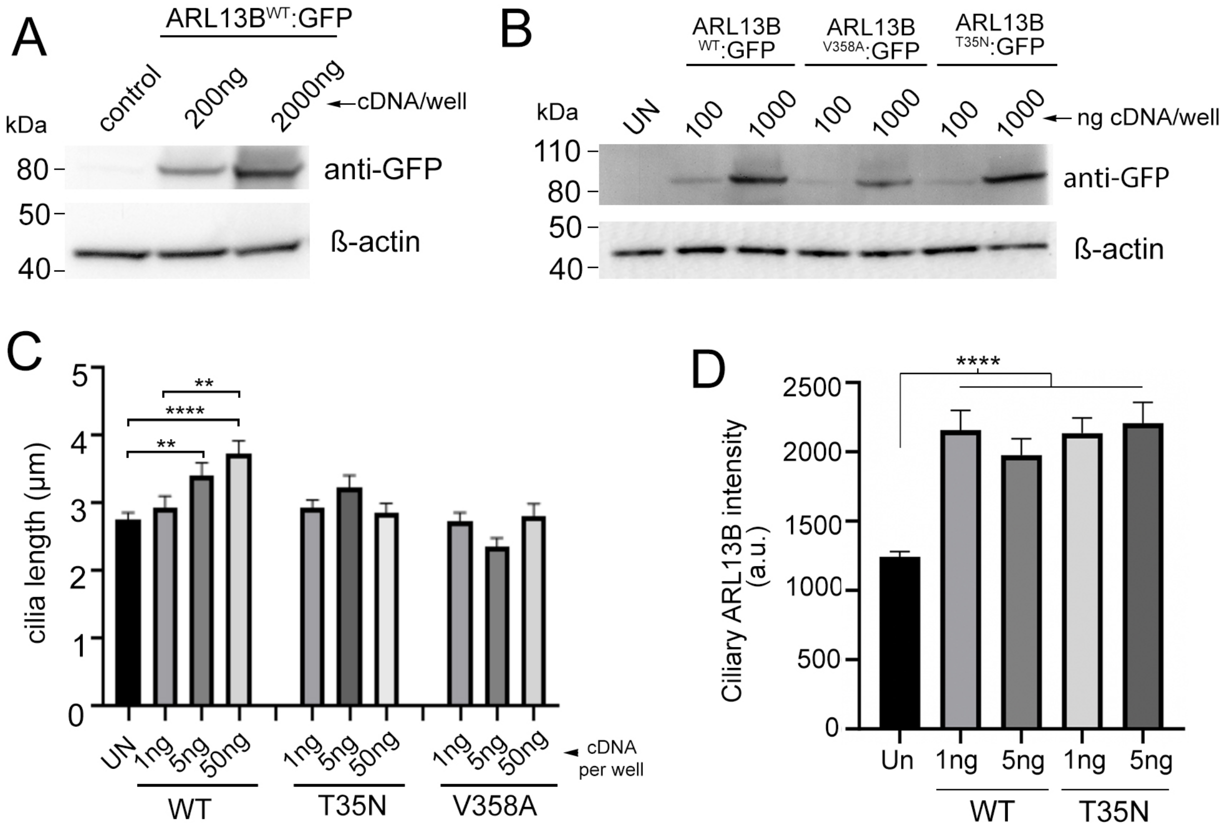

Figure S2. Effects of increasing WT and mutant *ARL13B:GFP* cDNA on ARL13B:GFP protein level, cilia length and ciliary ARL13B intensity. (**A**) S7 parental cells either untransfected (control) or with increasing concentrations (ng/well) of ARL13B^{WT}:GFP cDNA. Cells were harvested and lysed after 24hr and western blotted for GFP and ß-actin. (**B**) Control or increasing concentrations of ARL13B^{WT}:GFP, ARL13B^{V358A}:GFP, or ARL13B^{T35N}:GFP cDNA. Twenty four hours post-transfection, western blot for GFP and ß-actin. (**C**) Parental S7 cells were transfected with indicated concentration of ARL13B^{WT}:GFP, ARL13B^{T35N}:GFP, or ARL13B^{V358A}:GFP cDNA. Lengths of GFP^{+} cilia (n= 22-47 per group) for WT/T35N cells were measured after 48hr and compared to endogenous ARL13B^{+} cilia (n=34) for untransfected (UN) and V358A cells. **p<0.01, ****p<0.001 (ANOVA). (**D**) Quantification of the total ARL13B fluorescence intensity within cilia of untransfected (UN) (n= 34 cilia), 1ng WT (n= 22 cilia) 5ng WT (n= 31 cilia), 1ng T35N (n= 40 cilia), and 5ng T35N (n= 30 cilia) cDNA/well. **p<0.01, ****p<0.001 (ANOVA).

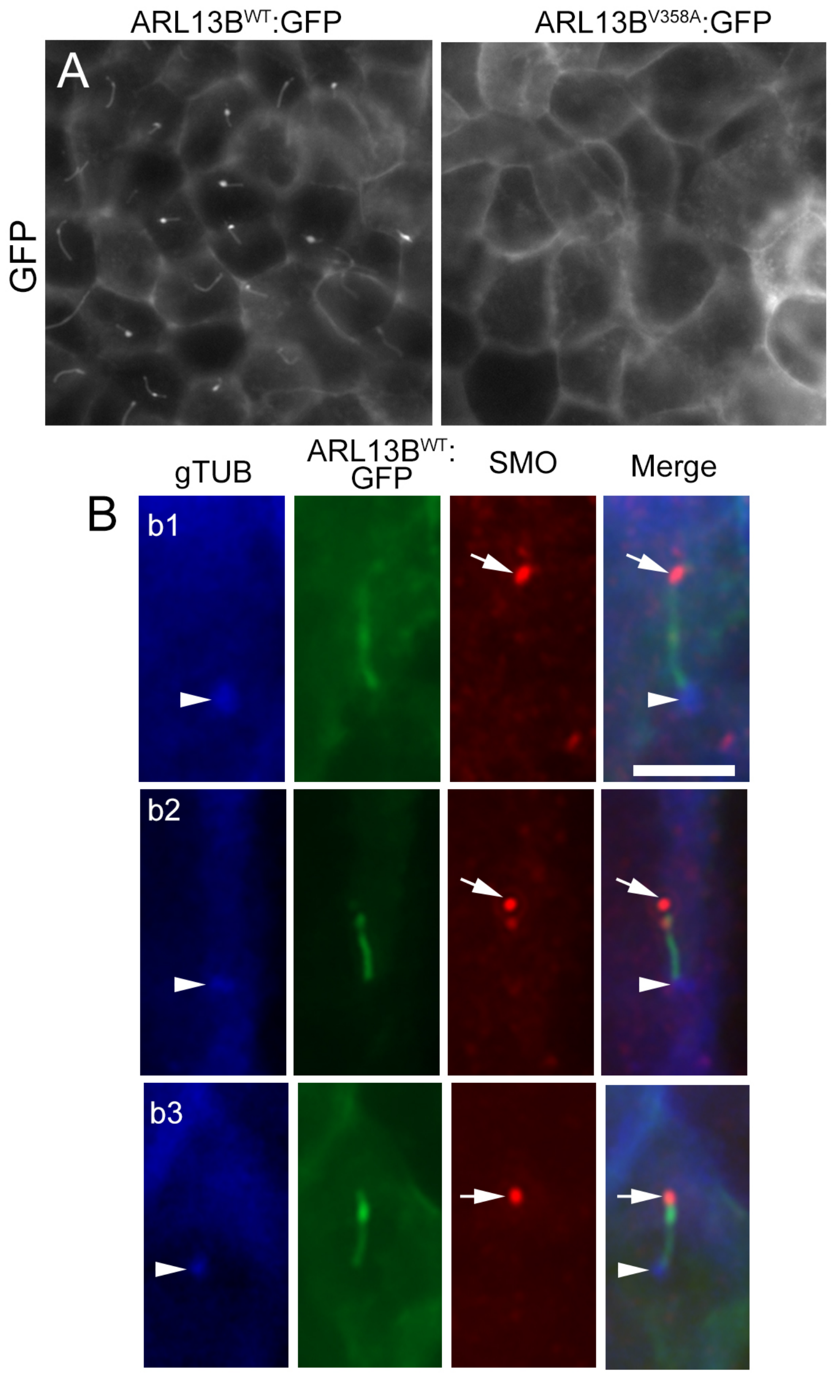

Figure S3. ARL13B^{WT}:GFP and ARL13B^{V358A}:GFP expression, and SMO localization on ARL13b^{WT}:GFP^{+} cilia. A) GFP channel alone in S7 cells from Figure 2A,B. **B)** Several examples of immunostaining for endogenous SMO (red, arrows) at the distal tips of ARL13B^{WT}:GFP+ cilia (green) whose ciliary basal bodies are gamma-tubulin+ (gTUB, blue, arrowheads). Scale bar = 5um.

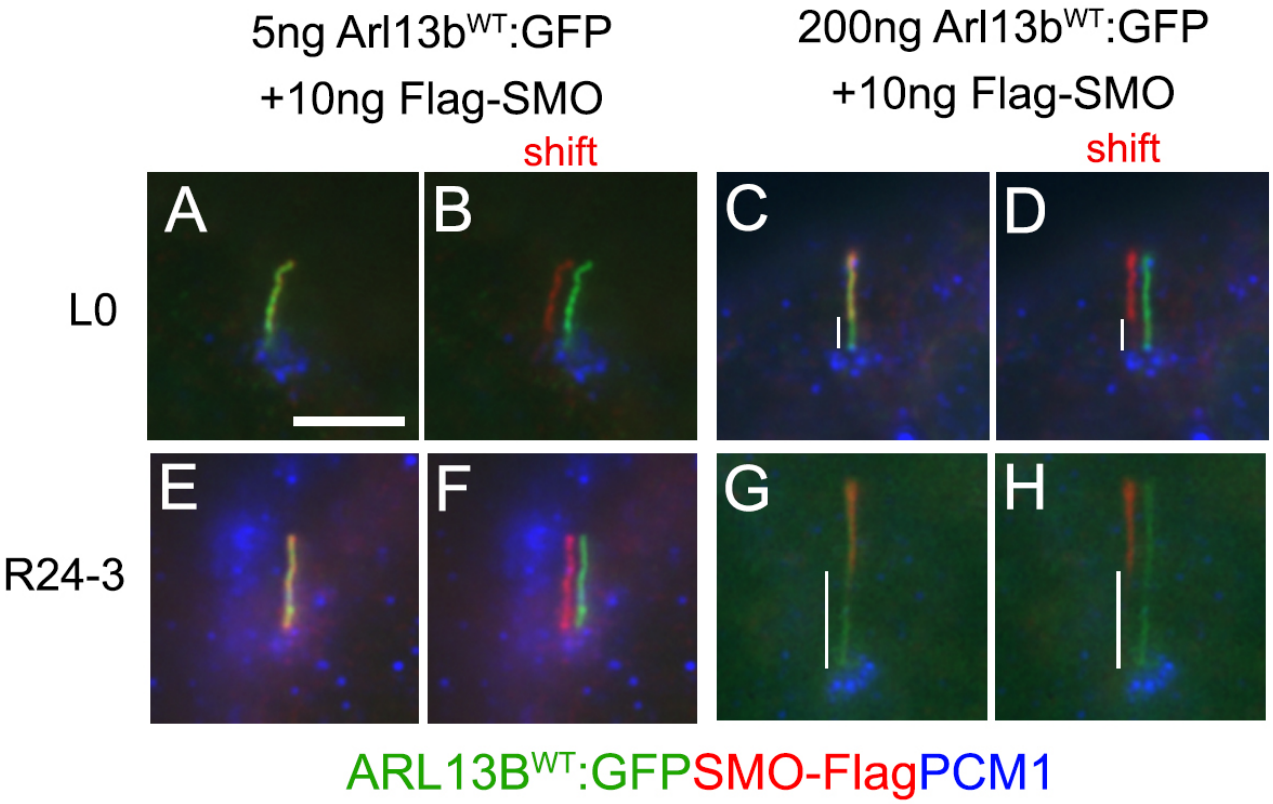

Figure S4. **Increasing ARL13B expression drives Flag-tagged Smo toward the ciliary tip.** Increasing ARL13B:GFP cDNA in human GBM lines L0 (**A-D**) or R24-3 (**E-H**) while holding the amount of Smo-Flag cDNA constant shows Smo-Flag beginning to accumulate toward the tip of cilia transfected with higher ARL13B:GFP cDNA. The cDNA amounts are ng/well. The shift panels in B, F, D and H have the SMO-Flag signal/channel shifted slightly left of the ARL13B^{WT}:GFP^{+} cilium. The white vertical lines in C, D, G, and H indicate proximal regions of the cilium displaying weak label for Smo-Flag compared to the more distal shaft of the cilium. Immunolabeling for PCM1 (blue) reveals the pericentriolar material that usually concentrates around the ciliary base. Scale bar in A = 5μm.

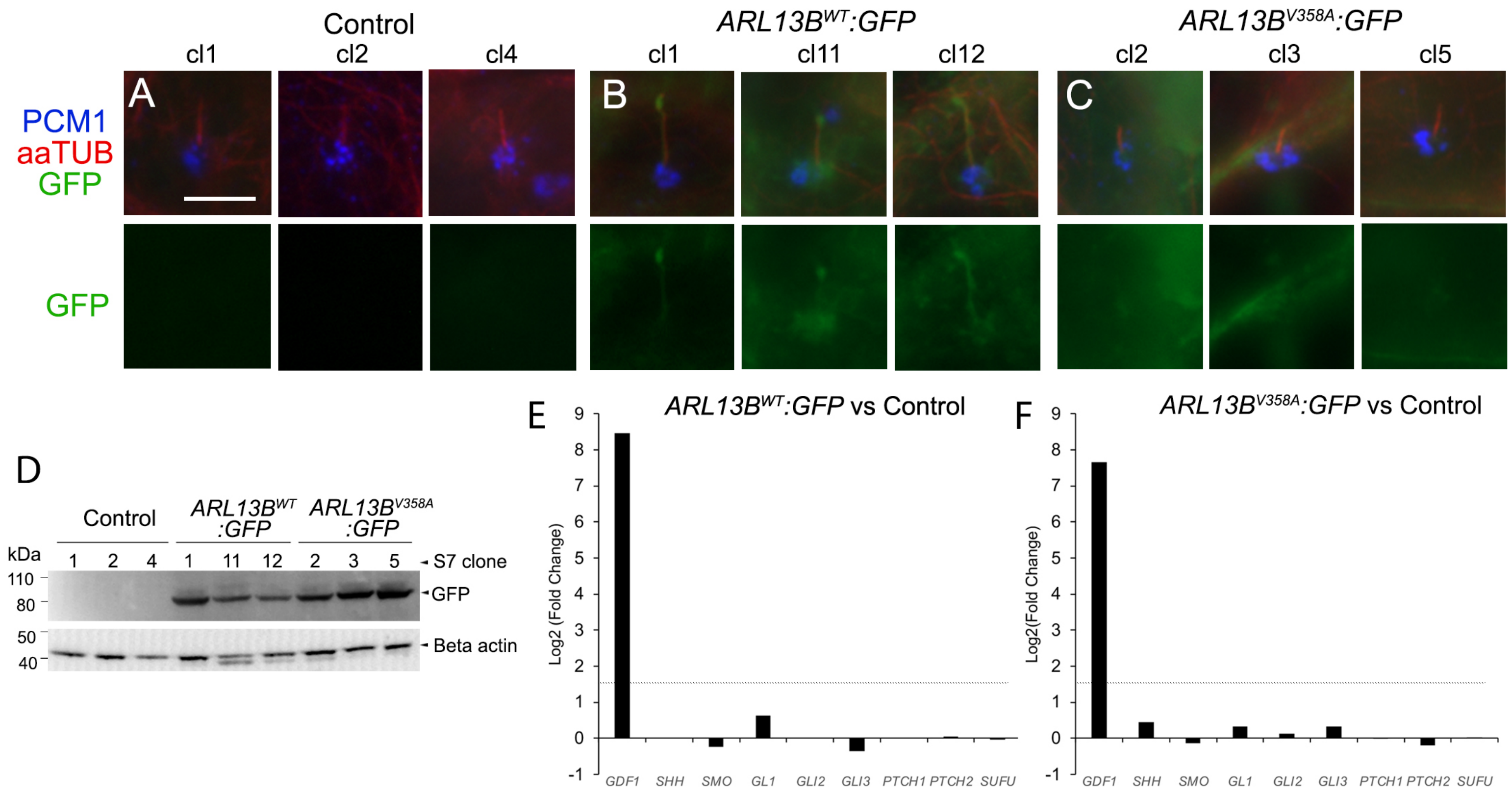

Figure S5. **Lack of SHH pathway activation in S7 *ARL13B^{WT}:GFP* and *ARL13B^{V358A}:GFP* transgenic cell lines.** (**A-C**) Isolated and expanded S7 control clones (cl1, cl2, and cl4), ARL13B^{WT}:GFP clones (cl1, cl11, cl12) (**B**), and ARL13B^{V358A}:GFP clones (cl2, cl3, cl5) (**C**). All cells were immunostained for GFP (green), acetylated alpha tubulin (aaTUB, red) and PCM1 (blue). Scale bar in A = 5µm. (**D**) Western blot shows GFP expression in WT and V358A expressing clones but not control clones. ß-actin is a loading control. (**E, F**) The clones in A-C were lysed and sent for bulk RNAseq analysis. Data from each WT clone was grouped and compared to grouped control clone data (**E**), and data from V358A clones were combined and compared to combined control clones (**F**). Compared to more highly expressed genes (e.g. *GDF1)* observed in both WT (**E**) and V358A (**F**) cell lines, genes in the SHH pathway fell below a 1.5 fold change threshold (dashed lines).

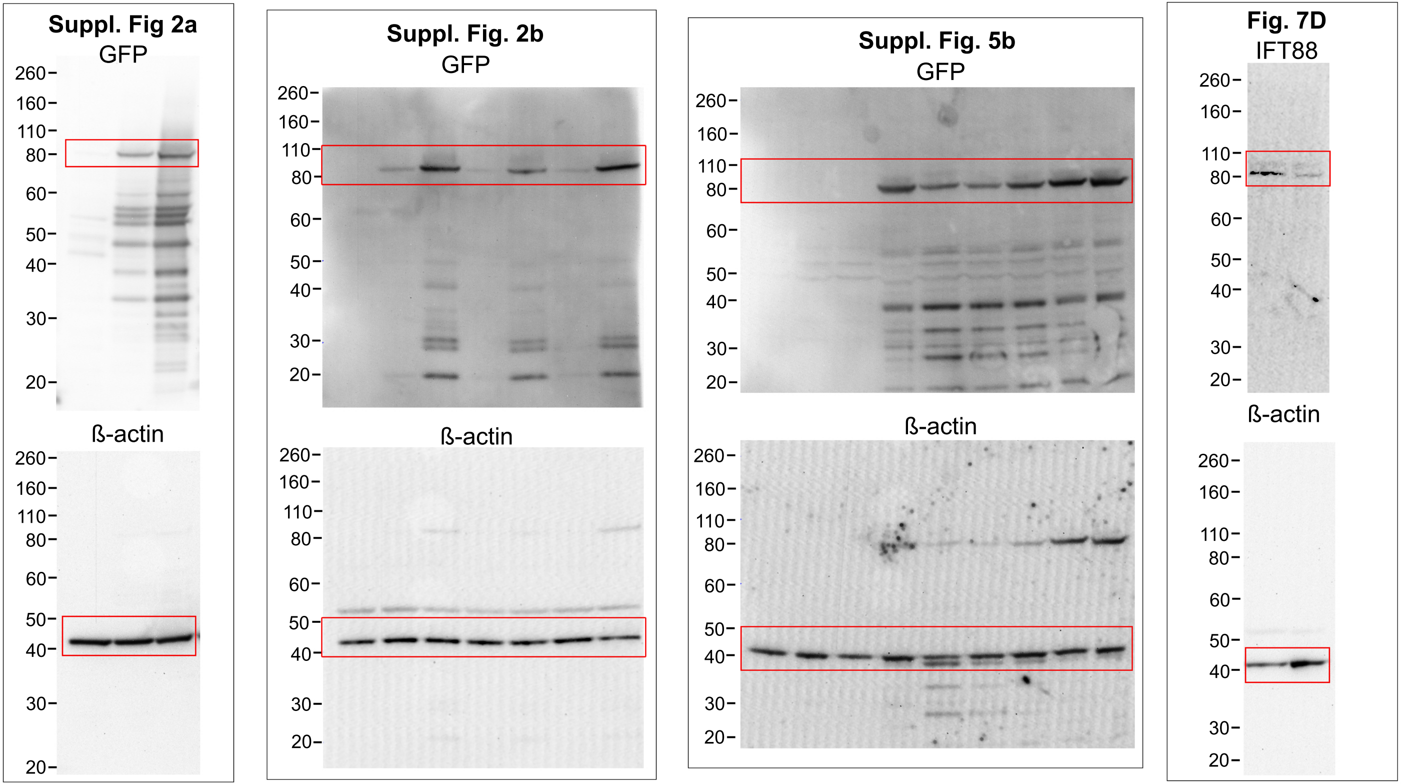
Suppl. Fig 2a
GFP
260
160
110
80
60
50
40
30
20
ß-actin
Suppl. Fig. 2b
GFP
ß-actin
Suppl. Fig. 5b
GFP
ß-actin
Fig. 7D
IFT88
ß-actin
